# Eicosanoid Biosynthesis in Male Reproductive Development: Effects of Perinatal Exposure to NSAIDs and Analgesic Drugs

**DOI:** 10.3389/ftox.2022.842565

**Published:** 2022-03-03

**Authors:** Amy Tran-Guzman, Martine Culty

**Affiliations:** Department of Pharmacology and Pharmaceutical Sciences, School of Pharmacy, University of Southern California, Los Angeles, CA, United States

**Keywords:** testis, NSAID, analgesics, testicular dysgenesis syndrome, perinatal exposure, infertility

## Abstract

Increasing rates of infertility associated with declining sperm counts and quality, as well as increasing rates of testicular cancer are contemporary issues in the United States and abroad. These conditions are part of the Testicular Dysgenesis Syndrome, which includes a variety of male reproductive disorders hypothesized to share a common origin based on disrupted testicular development during fetal and neonatal stages of life. Male reproductive development is a highly regulated and complex process that relies on an intricate coordination between germ, Leydig, and Sertoli cells as well as other supporting cell types, to ensure proper spermatogenesis, testicular immune privilege, and endocrine function. The eicosanoid system has been reported to be involved in the regulation of fetal and neonatal germ cell development as well as overall testicular homeostasis. Moreover, non-steroidal anti-inflammatory drugs (NSAIDs) and analgesics with abilities to block eicosanoid synthesis by targeting either or both isoforms of cyclooxygenase enzymes, have been found to adversely affect male reproductive development. This review will explore the current body of knowledge on the involvement of the eicosanoid system in male reproductive development, as well as discuss adverse effects of NSAIDs and analgesic drugs administered perinatally, focusing on toxicities reported in the testis and on major testicular cell types. Rodent and epidemiological studies will be corroborated by findings in invertebrate models for a comprehensive report of the state of the field, and to add to our understanding of the potential long-term effects of NSAID and analgesic drug administration in infants.

## 1 Introduction

The recent decades have seen concerning increases in male reproductive disorders and diseases. Total fertility rates have been trending downward in places such as Europe, Japan, and United States, and this decline has been correlated with increases in testicular dysgenesis syndrome (TDS). TDS includes a variety of male reproductive disorders such as infertility, poor semen quality, cryptorchidism, and hypospadias, which are hypothesized to share a common origin of disrupted testicular development during fetal and neonatal stages of life (Skakkebaek et al., 2016). A study evaluating cancer rates of a Danish registry found that testicular cancer rates have more than tripled between 1944–2009 (Andersson et al., 2016). Furthermore, cryptorchidism, or undescended testes, is one of the most commonly diagnosed birth defects (Boisen et al., 2004). Overall, negative trends in global sperm counts and decreases in fertility rates (Mehta et al., 2016; Levine et al., 2017) suggest that there is much more to learn about the biological factors that contribute to reductions in male reproductive capacity. One hypothesis is that lifestyle factors, such as greater exposure to environmental toxicants and pharmaceuticals in the modern world, may be contributing to the significant rates of TDS diagnoses in contemporary times (Skakkebaek et al., 2016).

Over-the-counter pharmaceuticals have been drawing greater attention recently as potential endocrine disrupting compounds (EDCs) to be concerned about, due to their widespread use and presumed safety for administration throughout life. One such class of drugs include cyclooxygenase (COX) inhibitors or non-steroidal anti-inflammatory drugs (NSAIDs), which are widely prescribed for pain relief and fever reduction. Acetaminophen (paracetamol), a non-specific COX inhibitor used for its analgesic and antipyretic properties, was reported to be used during pregnancy in 62% of woman surveyed (Bandoli et al., 2020), and ibuprofen, an NSAID, was also found to be used commonly during pregnancy (Kristensen et al., 2011). However, a growing body of evidence suggests that *in utero* exposures to such drugs have the potential of altering reproductive development during fetal stages and subsequent reproductive function in the offspring. Maternal exposure to common NSAIDs and analgesic drugs during pregnancy was found to be significantly positively associated with cryptorchidism in infants (Berkowitz and Lapinski 1996; Jensen et al., 2010), specifically in the second trimester (Kristensen et al., 2011). Currently, the US Food and Drug Administration is recommending against taking NSAIDs at 20 weeks or later during pregnancy, due to their ability to cause kidney problems in unborn babies (FDA 2020), but no recommendation has been made against the use of analgesic drugs such as acetaminophen during pregnancy or the use of NSAIDs in infants. In addition to *in utero* exposure, babies can be exposed to these drugs either in the form of direct administration or passed through breast milk from maternal ingestion after birth (Siu et al., 2000; Briggs et al., 2012). The first postnatal months represent a complex period during male reproductive development in which the population of spermatogonial stem cells (SSCs) is established to support lifelong spermatogenesis. Therefore, an assessment of how NSAID and analgesic drugs are impacting male reproductive development during the early stages of life is necessary to obtain a comprehensive understanding of their contributions to male reproductive diseases.

## 2 Fetal and Neonatal Male Reproductive Development in Rodents and Human

### 2.1 Main Cell Types and Timepoints in Testis Development

Gonadogenesis is a dynamic and finely tuned system, requiring the regulation of multiple molecular processes, many occurring simultaneously. The first sign of gonadogenesis is the thickening of the coelomic epithelium, forming the genital ridge. Germ cell development initiates with the commitment of embryonic stem cells to the germ cell lineage, forming primordial germ cells (PGCs) expressing characteristic germ cell signature genes (Culty 2009). From gestational day (GD)7.5–13 in rodents, sexually undifferentiated PGCs, upon receiving Kit and CXCR4 signals, migrate and become resident at the genital ridge. PGCs undergo a genomic erasure process in which parental DNA methylation patterns are removed (Spiller and Bowles 2015). Sex determination occurs on GD12.5, driven by Sry expression in male somatic cells which differentiate into fetal Sertoli cells, whose main function is to assist germ cell development and spermatogenesis. At this stage, fetal Sertoli cells committed to epithelial differentiation organize intimately around the germ cells, which are now considered fetal gonocytes (pro/pre-spermatogonia). Sertoli cells drive the organization of the seminiferous cords as well as the recruitment and differentiation of mesonephros-derived cells to peritubular myoid cells located at the outer periphery of the cords, and fetal Leydig cells, the steroidogenic cells found in the interstitium surrounding the cords. Cells derived from the mesonephros give rise to endothelial cells that contribute to the formation of blood vessels and support the structure of the cords. Both Sertoli and peritubular myoid cells contribute to the formation of the basement membrane surrounding the seminiferous cords. Leydig cells are primarily responsible for the production of male sex hormones, mainly testosterone and dihydrotestosterone, that regulate male sex differentiation, the development of internal and external genitalia, as well as secondary sex characteristics. Male germ cells from fetal to juvenile phases of development are prevented from entering meiosis through the degradation of retinoic acid (RA) by cytochrome CYP26B1 and NANOS2 expression (Li et al., 2009; Spiller and Bowles 2015).

Fetal Sertoli cells in humans double every 2 weeks from GW7-19 and continue to amplify until cell numbers peak at birth. After birth, Sertoli cell numbers remain stable until puberty where the cells reach a final count of approximately 1800 × 10^6^ cells/testis. Leydig cells are first detected in humans at GW 8 and cell numbers increase exponentially until GW18. Thereafter, a dedifferentiation process occurs and Leydig cell numbers decline in the fetus until after birth when numbers increase up to 3 months postnatal, likely due to increases in LH levels. The cells then decline at 4 months and are scarce until age six to 8 years, at which point they increase to reach adult levels of approximately 800 × 10^6^ cells/testis. Germ cell numbers in humans exponentially increase from the initiation of testis differentiation to the end of the second trimester, and is greatest from GW6-10, at which point it decreases until the second trimester. Asynchronous development of undifferentiated gonocytes occurs throughout fetal and neonatal stages of development, followed by a marked increase of undifferentiated spermatogonial including SSCs between birth and puberty (Plant and Zeleznik 2014).

In rodents and humans, early male germ cell development comprises phases of quiescence, proliferation, migration and differentiation (Manku and Culty 2015). Apoptotic processes also play integral roles by ensuring removal of defective cells and maintaining proper germ-Sertoli cell ratios. Sertoli cells assist with the maturation of neonatal gonocytes into spermatogonial stem cells (SSCs), which will support lifelong spermatogenesis. At postnatal day (PND)3 in rats, gonocytes re-enter mitosis and migrate towards the basement membrane of the seminiferous cords, where they undergo differentiation into SSCs or Type A spermatogonia of the first spermatogenic wave around PND6-8. In human, testis cords begin to form between the seventh and ninth gestational week (GW). Formation of the essential structure of the testis takes approximately 8 weeks, leading to establishment of major testicular cell types (Gaskell et al., 2004). The cords give rise to seminiferous tubules and the differentiation of immature Sertoli cells into androgen-responsive cells leads to the formation of an intratubular lumen. Tight junction proteins organize between Sertoli cells into the testis-blood-barrier, a crucial structure to maintaining testicular immune privilege (Piprek 2016).

The rodent spermatogenic cycle involves distinct phases along the seminiferous tubules taking place simultaneously: 1) mitosis in SSCs to type B differentiated spermatogonia; 2) spermatogonial differentiation in which SSCs mature from undifferentiated progenitors to differentiated spermatogonia; 3) the differentiation of successive types of spermatocytes and a lengthy meiotic phase; 4) the formation of haploid spermatids during spermiogenesis, a metamorphosis phase ultimately forming spermatozoa released into the lumen during spermiation. Several steps of postnatal germ cell differentiation and the entry to meiosis are regulated by Sertoli cell-produced RA (Griswold 2016). The human spermatogenic cycle is similar. Type A spermatogonia, comprising of dark type A and pale type A spermatogonia, undergo mitotic proliferation and differentiate to Type B spermatogonia. Type B spermatogonia further differentiate to spermatocytes (leptotene, zygotene, pachytene, and diplotene), which are present upon puberty in humans. Diplotene spermatocytes undergo meiosis I to form secondary haploid spermatocytes and further undergo morphological transformations from round spermatids to spermatozoa (Chen et al., 2017).

Another type of cell that plays multiple roles in testis development and adaptation to its environment are the testicular macrophages, known to exert not only immune functions, but also to modulate Leydig and germ cell functions. While rodent primitive macrophages are first seen in gonadal primordium, as early as E10.5 in mice, fetal rat macrophages have been observed in testis starting at GD16 to 19. Rodent fetal testicular macrophages express mainly M2 polarization-type markers and are believed to be important for maternal-fetal tolerance (Meinhardt et al., 2018; Gu et al., 2021). The numbers of macrophages increase slowly until the onset of puberty, where macrophages rapidly increase and decline thereafter. During puberty, testicular macrophages interact intimately with Leydig cells through digitations or microvillus-like processes that are inserted within coated pits of the macrophages (Hutson 1990; Hutson 1992; Plant and Zeleznik 2014; Gu et al., 2021).

Animal studies, including ours, have established the susceptibility of the fetal and neonatal testis to environmental insults, leading to short- and long-term adverse effects in testicular cells, including germ cells (Foster 2006; Jones et al., 2015; Walker et al., 2020; Walker et al., 2021). Several groups have theorized that testicular germ cell tumors (TGCTs) may arise from the disruption of SSC formation resulting from the vulnerability of PGC or gonocyte populations to environmental exposures (Sharpe 2006; Rajpert-de Meyts and Hoei-Hansen 2007). Therefore, there is great interest in understanding the molecular mechanisms driving the establishment of the SSC pool, with the goal of determining the origins of disrupted spermatogenesis and/or TGCT formation. Furthermore, a greater understanding of the etiology of TDS can lead to developing interventions to rescue the SSC population (Kanatsu-Shinohara et al., 2004; Guan et al., 2006; Izadyar et al., 2008).

### 2.2 Testicular Steroidogenesis

The steroidogenic pathway is generally well-conserved between rodents and humans, but there are several differences. In early fetal Leydig cells, androgen production occurs independently of the pituitary, and it is only in late fetal age that testosterone production becomes LH-dependent (Habert et al., 2001). In the mouse, studies have shown that LH is not required for fetal Leydig cell to produce testosterone, but rather fetal Leydig cells produce androstenedione that is converted to testosterone by fetal Sertoli cells, a fact recently supported by Single-cell RNA-seq analysis of fetal and adult mouse Leydig cells (Shima et al., 2013; Sararols et al., 2021). In rats, fetal Leydig cell development is largely independent of LH, but become LH-dependent few days before birth (O’shaughnessy et al., 1998; Habert et al., 2001; Zhang et al., 2004). In humans, fetal Leydig cells are critically dependent upon stimulation of LH throughout most of fetal development.

Testosterone synthesis also differs between rodent and humans. Mice and rats synthesize testosterone through the Δ^4^ pathway, whereas humans predominantly use the Δ^5^ pathway. In the Δ^4^ pathway, pregnenolone is converted to all Δ^4^ steroids by HSD17B and the final step in testosterone synthesis, the reduction of androstenedione, is also catalyzed by HSD17B in fetal Leydig cells. However, this process does not completely rely on Leydig cells, as androstenedione can also be converted by Sertoli cells in mice lacking HSD17B (O’Shaughnessy et al., 2000; Shima et al., 2013). Human Leydig cells synthesize testosterone using the Δ^5^ pathway, in which the first reaction is catalyzed by CYP17A1 (Li et al., 2018). Often more than one HSD enzyme can catalyze the same reaction. For example, the reduction of androstenedione to testosterone can involve both ARK1C3 and HSD17B (Plant and Zeleznik 2014).

### 2.3 Disruption of Testis Development

As described in previous sections, the development of the male reproductive system relies on the formation of a functional fetal testis, the sequential production of hormones from fetal Sertoli and Leydig cells, and the establishment of a pool of spermatogonial stem cells from perinatal germ cell precursors in early postnatal ages. The disruption of these processes leads to conditions that define the Testicular Dysgenesis Syndrome (TDS). In addition to testis cancer, TDS includes cryptorchidism, hypospadias, altered testosterone levels, poor semen quality, and reduced anogenital distance (AGD). Cryptorchidism is one of the most common birth defects, corresponding to the failure of one or both testes to descend to the scrotum before birth. If not corrected, the retention of testes in the abdomen results in the loss of spermatogenic cells in early childhood, and increased risks of infertility or TGCTs, whereas corrective surgery *via* early orchidopexy can reduce chances of developing such reproductive disorders. Hypospadias, which is due to androgen deficiency in the fetus, is diagnosed when the urethra opens on the ventral side of the penis instead of the tip, which may require surgical reconstruction of the penile urethra in severe cases (Skakkebaek et al., 2016). Postnatally, testosterone is important for sustaining secondary male sex characteristics and spermatogenesis. The trend towards lower testosterone levels observed in the US and Europe is believed to be related to the decline in male reproductive health (Travison et al., 2007). As testosterone is the major paracrine messenger involved in crosstalk between testicular cells, compromised testosterone levels can affect testicular function. Semen quality has served as a measurement for male fertility by the World Health Organization (WHO 2010). Low sperm count and quality (motility and morphology) have been associated with decreasing rates of conception on a population-wide level (Guzick et al., 2001). In males, the AGD is 50–100% longer than in females (Sathyanarayana et al., 2010). Shorter AGDs and smaller penis size reflect decreased fetal androgen levels (Thankamony et al., 2014; Skakkebaek et al., 2016). Reduced AGD has been found in men in association with cryptorchidism, low sperm counts, and hypospadias, further supporting the common origin of these disorders. Epidemiological studies have also reported strong correlations between shorter AGD in children and the levels of phthalate plasticizers measured in maternal urine (Bornehag et al., 2015).

The testis is a complex organ that requires coordinated crosstalk between several major cell types for normal function. Fetal development and infancy represent periods of dynamic growth and maturation of various cell types within the testes and disrupting these processes may lead to long-term reproductive diseases and syndromes. Due to the similarities in male reproductive development and the regulation of steroidogenic pathways between human and rodents, rodent models are useful tools in studying specific mechanistic effects of NSAID and analgesic drugs on the male reproductive system. However, to ultimately translate these findings to real-life applications, human-derived models and epidemiological studies must be considered. Therefore, this review will weigh evidence from both rodent and human models, to provide a comprehensive overview of the field.

## 3 Eicosanoid Biosynthesis and COX Inhibitors

### 3.1 Enzymes of the Eicosanoid Pathway

**FIGURE 1:**
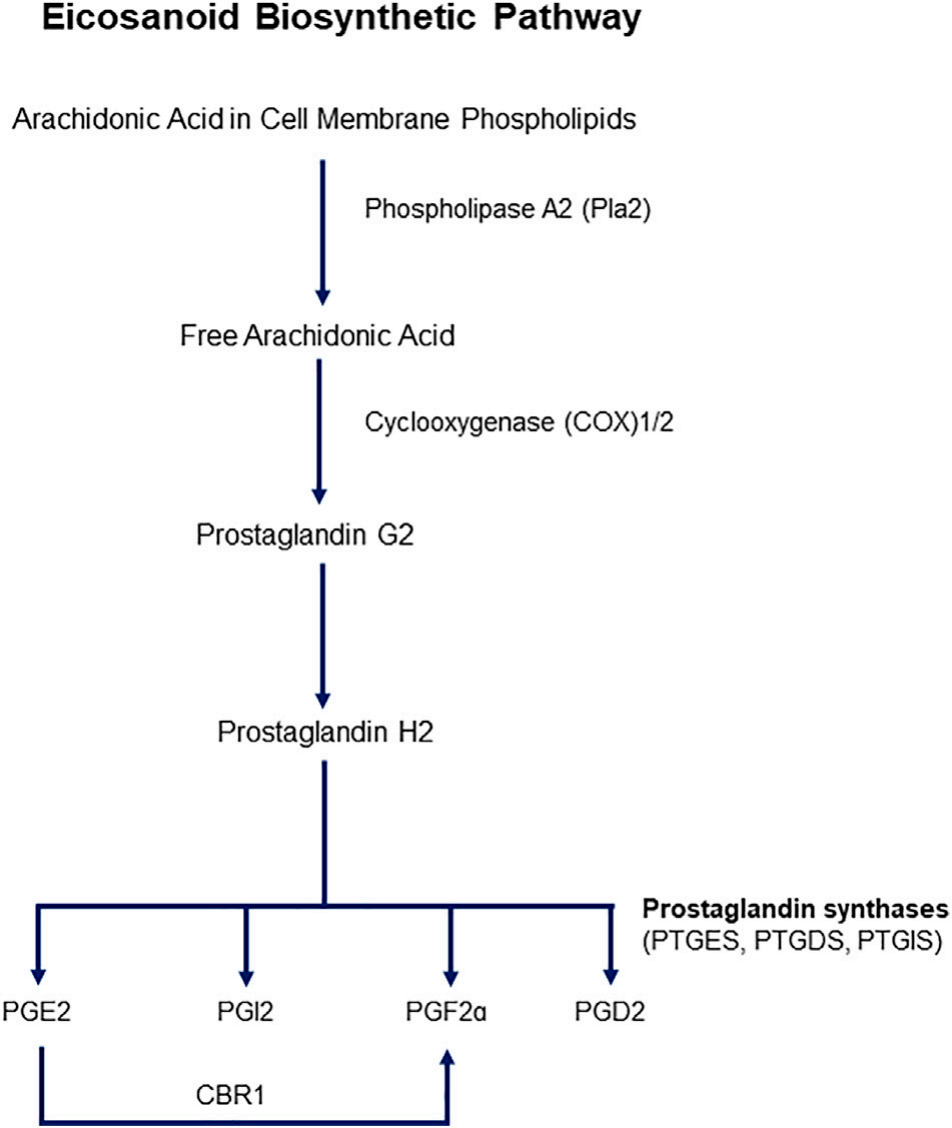
Schematic representation of the eicosanoid pathway. Abbreviations: PG -E2, -I2, -F2a, -D2: Prostaglandins -E2, I2, F2a, D2. CBR1: Carbonyl reductase 1. PTGES: Prostaglandin E Synthase, PTGDS: Prostaglandin D Synthase, PTGIS: Prostaglandin I Synthase.

Cyclooxygenases are enzymes that catalyze the first two steps in the biosynthesis of prostaglandins (PGs) from arachidonic acid (AA). The main steps of the pathway are illustrated in Figure 1. Two isoforms of COXs have been characterized, identified as COX1 and COX2. COX1 is constitutively expressed, and COX2 is inducible upon an external stimulus. Both isozymes are 71 kDa in molecular weight and are almost identical in length, with over 63% similarity in amino acid sequence. AA is the major prostanoid precursor and both isoforms of COX enzymes oxygenate this substrate through identical enzymatic processes (Vane et al., 1998; Smith et al., 2000). The release of AA from membrane-bound phospholipids is catalyzed by phospholipase A2 (PLA2), of which there are several isoforms that may be expressed constitutively or can appear after inflammatory insult (Cirino 1998). The biosynthesis of prostanoids requires a 3-step enzymatic process: 1) Stimulus-initiated hydrolysis of AA from glycerophospholipids involving secretory or cytoplasmic PLA2 (sPLA2, cPLA2), 2) oxygenation of AA by the COXs to yield PGG2 and PGH2, and 3) conversion of PGH2 to biologically active prostaglandins PGs (PGD2, PGE2, PGF2a, PGI2) and thromboxane (TXA2). Thereafter, the prostanoid end products exit the cells to activate G-protein-coupled prostanoid receptors, or in some cases interact directly with nuclear receptors (Smith et al., 2000).

PG synthases are responsible for the enzymatic conversion of PGH2 to the major prostanoids, which are isoform specific. PGD synthase (PGDS/PTGDS) catalyzes the conversion of PGH2 to PGD2, PGE synthase (PGES) to PGE2, PGFa synthase to PGF2a, and PGI synthase (PGIS) to PGI2, or prostacyclin (Smith and Murphy 2016). Two forms of PGDSs have been characterized, glutathione-dependent and -independent. Glutathione-dependent hematopoietic PGDS (H-PGDS) exhibits glutathione-S-transferase activity, whereas lipocalin PGDS (L-PGDS) conversion of PGH2 to PGD2 does not require this transferase activity. Three PGESs have been studied and all require reduced glutathione as a cofactor-cytosolic PGES (cPGES), an inducible, microsomal PGES (mPGES-1), and a non-inducible microsomal PGES (mPGES-2) (Murakami and Kudo 2004). Formation of PGF2a involves first a reduction of PGH2 and a PGF_2_S catalyzing NADPH, and PGF2a prostanoid that requires an NADPH-dependent synthase. PGF2a can also be synthesized from PGE2 by carbonyl reductase 1. PGI synthases are believed to be localized to the cytosolic side of the endoplasmic reticulum (ER), therefore PGH2 formed in the lumen of the ER can diffuse across the membrane to be converted to PGI2 on the cytosolic side of the membrane.

Though similar in structure and function, and often co-expressed in the same cell, the differences between the two COX isozymes lie in their unique downstream signaling properties (Smith et al., 2000). COX2 will accept a wider range of fatty acids than COX1, and has the ability to oxygenate other substrates in addition to AA, such as eicosapentaenoic acid, y-linolenic acid, a-linolenic acid and linoleic acid (Vane et al., 1998). Stimuli known to induce COX2 expression are those associated with inflammation such as pro-inflammatory cytokines (IL-2, IL-1, and TNFa) and lipopolysaccharide (LPS), whereas anti-inflammatory cytokines (IL-4, IL-10, and IL-13) can decrease the induction of COX2 (Vane et al., 1998). As a constitutively expressed enzyme, COX1 is important for maintaining the homeostasis or “housekeeping” roles of the body, such as providing PGs to the stomach and intestine to maintain the mucosal epithelium. COX1 also plays a crucial role during development by synthesizing PGs that are responsible for the survival of fetuses, such that fetuses born from homozygous COX1 knockout mice did not survive past birth (Langenbach et al., 1995). It is hypothesized that their unique properties are derived from their expression: COX1 being involved in producing PGs that act extracellularly to mediate responses to external signals, while the ability of COX2 to be induced suggests also a role in augmenting the function of COX1 upon an inflammatory response (Smith et al., 2000).

### 3.2 Prostaglandins

PGs are important lipid mediators that have widespread roles based on their unique chemical structures, but are mainly involved in inflammatory responses (Seo and Oh 2017). They bind and act *via* specific G-coupled protein receptors (GPCRs) (Mitchell and Warner 1999). PGI2 bind to IP receptors that lead to cAMP elevation, generally leading to an inhibition of many cellular processes. PGE2 can bind on one of four isoforms of EP receptors (EP1-4) that are each linked to different signal transduction pathways that can either activate or inhibit cellular responses (Pierce and Regan 1998). PGD2 and PGF2a bind to DP and FP receptors, respectively, which are linked to activation of inositolphosphates and subsequent increase in intracellular calcium, and usually result in activation of cellular processes. Besides mediating varied local cellular responses, the action of PGD2 and PGF2a on calcium signaling is critical in females for ovulation and during gestation and parturition. Indeed, the eicosanoid system plays an important role during pregnancy. The COX enzymes are expressed in the uterine epithelium at different times during pregnancy and are involved in regulating feedback mechanisms associated with embryogenesis (Chakraborty et al., 1996). COX1 is expressed in the uterine epithelium prior to implantation, whereby thereafter it gets downregulated. Subsequently, COX2 gets expressed, suggesting a possible role of COX2 in maintaining blastocyst attachment. During labor and delivery, PGs (primarily PGE2 and PGF2a) are involved in smooth muscle contraction of the uterus and cervical ripening (O'Brien 1995). Due to the significant involvement of PGs to induce contractions during labor, NSAIDs have been prescribed to delay premature labor by inhibiting the production of PGs (Sawdy et al., 1997). These cellular responses are dependent on the target tissues and cells of which the PG receptors are expressed.

### 3.3 Pharmacology of NSAIDs and Analgesic Drugs

The main pharmacology of NSAIDs is to inhibit COX activity and subsequent PG production, thereby exerting anti-inflammatory and pain relief properties (Mitchell and Warner 1999). The unique mechanisms of COX inhibition and drug preference to specific COX isozyme are dependent on the drug classes. Classical NSAIDs developed prior to 1995 have ability to inhibit both isozymes, but in general bind more tightly to COX1 (Laneuville et al., 1994). The inhibition of COX by aspirin is due to irreversible acetylation of the active site on the enzyme, leaving the peroxidase site unaffected (Vane et al., 1998). Other NSAIDs, such as ibuprofen or indomethacin, can produce either reversible or irreversible inhibition by competition with AA for the active site of the enzyme. More modern NSAIDs were developed to target specifically COX2 by taking advantage of a hydrophobic side pocket in its main channel, which is inaccessible in COX1 due to steric hindrance of the presence of an isoleucine rather than a valine at position 523 (DeWitt 1999; Marnett et al., 1999). The main concerns of non-selective COX inhibitors is due to their ability to also target COX1, which is highly expressed in the stomach and intestine (Smith et al., 2000).

Acetaminophen, for example, has a greater selectivity for COX2 than for COX1, and share many properties of COX2 selective inhibitors such as minimal gastric side effects (Hinz et al., 2008). However, in contrast to other NSAIDs, COX inhibition by acetaminophen is dependent on levels of cellular peroxides, and only at low cellular peroxide levels does acetaminophen inhibit the production of PGs. In contrast, selective COX2 inhibitors like celecoxib are effective anti-inflammatory agents in diseases like rheumatoid arthritis where peroxide levels are high as they are not peroxide dependent (Ouellet and Percival 2001; Graham et al., 2013).

As little is currently known about the contribution of perinatal exposure of pharmacological COX inhibitors to later life testicular disorders, this review will aim to take a deep dive into the involvement of the eicosanoid system in the regulation of fetal and neonatal germ cell development, as well as its role in overall testicular homeostasis. We will provide an overview of the eicosanoid biosynthetic pathway, explore its expression and function in developing and healthy mammalian testes, and evaluate the impact of NSAID and analgesics exposure on cells and processes related to male reproductive development and function. The current body of knowledge on the role of COXs and PGs in male reproductive systems, in conjunction with the toxicities reported with exposure of pharmacological COX inhibitors during early male reproductive development will be discussed in detail. These rodent and epidemiological studies will be corroborated by findings in non-rodent models for a comprehensive report of the state of the field, and to add to our understanding of long-term effects of NSAID and analgesic drug administration to infants.

## 4 Eicosanoid System in Male Reproductive Development

### 4.1 Expression and Role of Cyclooxygenases

While it is well established that the eicosanoid cascade plays a large role in the female reproductive system, particularly in labor and childbirth, less is known about the involvement of this pathway in the male reproductive system. Early efforts to establish roles of COX enzymes in male fertility using knockout mouse models determined that male fertility was not affected in COX1 or COX2 null mice, whereas COX2 null female mice were infertile (Lim et al., 1997). Thus, it was presumed early on that PGs do not play a role in male reproduction, and there was little interest in evaluating this further. More recently, however, interest in the involvement of the eicosanoid pathway in male reproduction peaked again and studies started to contradict or refine this conclusion. In 2003, Hase et al. found that while there was no expression of COX1 and COX2 in normal human testes, these enzymes were highly expressed in men with testicular cancer. Upon investigation of COX inhibitors on human testicular cancer cell lines (NEC-8), they found weak inhibition of cell viability with both COX1 and COX2 selective inhibitors, suggesting a role in of COX enzymes in promoting growth of testicular cancer cells (Hase et al., 2003). Another study reported expression of COX2 in interstitial cells in human testes biopsies of infertile male patients with Sertoli-cell-only syndrome (SCO-Syndrome) and patients with germ arrest (GA) syndrome (Frungieri et al., 2002). In 2010, Matzkin et al. reported that a reason for the upregulation of COX enzymes in infertile patients may be in response to action of proinflammatory cytokine IL-1. They also reported higher levels of PGD2 and PGF2a in the testes of these men (Matzkin et al., 2010). It appears that pathological human testes and those with altered morphology are ones that express COX enzymes and can produce PGs, suggesting a role of COX enzymes in the pathogenesis of testicular diseases.

The dysregulation of COX enzyme expression in pathological conditions can suggest that these enzymes may have roles in maintaining the normal functioning of the testes, and therefore efforts have been made to determine baseline levels of expression of COX enzymes in different cell types of the organ. In 2007, COX1 and COX2 mRNA was detected in both normal and LPS conditions of rat testes in somatic cells (Leydig and Sertoli cells) and testicular macrophages, as well as measurable amounts of PGE2 (Winnall et al., 2007). They reported that COX2 expression was greater than that of COX1 and treatment with a COX2 specific inhibitor, NS398, suppressed testicular PGE2 production that was not observed with a COX1 specific inhibitor. Detectable COX2 expression was also reported in Leydig cells in the human (Frungieri MB 2007) and the Syrian hamster (Matzkin et al., 2009). COX2 expression and PGs production were reported in hamster and rat Sertoli cells where its regulation by FSH and testosterone is proposed to play a role in glucose uptake and spermatogenic efficiency (Matzkin et al., 2012; Frungieri et al., 2015). COX1 is expressed in human testicular peritubular cells (HTPCs), which were also found to secrete PGE2 and express EP receptors (Rey-Ares, Rossi et al., 2018). Moreover, our lab recently reported the presence of COX enzymes and detectable levels of PGs in neonatal rat gonocytes (Manku et al., 2020) and in mouse and rat spermatogonia (Tran-Guzman et al., 2022). Detectable baseline levels of expression of COX enzymes and PGs in several models support a maintenance role of the eicosanoid system in male reproduction, however the exact mechanism behind this role remains elusive.

Several roles of COX enzymes have been considered to explain the positive correlation between COX expression and/or function with pathological testicular conditions. Reports have suggested COX involvement with steroid biosynthesis or feedback induction when spermatogenesis is impaired (Wang et al., 2003). More recently, Kubota et al. examined the potential role of COX2 in the pathogenesis of testicular cryptorchidism. In an experimental cryptorchidism mouse model, COX2 expression was induced in cryptorchid testes with 4-fold greater mRNA expression compared to controls, whereas COX1 expression remained unchanged (Kubota et al., 2011). Disturbance of the arrangement of cells in the seminiferous tubules suggested that spermatogenesis was disrupted by experimental cryptorchidism, leading to the proposition that COX2 may play a role in protecting germ cells against injury, a proposal which has yet to be confirmed by other studies. However, we cannot disregard the involvement of other processes contributing to the pathogenesis of cryptorchidism that may be targeted by COX inhibitors. The leading hypothesis is that COX inhibitors can act as endocrine disruptors and interfere with steroidogenesis, which will be elaborated in Section 4. Furthermore, the exposure of these drugs during the critical male programming window (GD15.5–18.5 in rats, GW8-14 in humans) may be involved in diminishing normal androgen production. Insulin like 3 (*INSL3*) expression in human fetal Leydig cells was also shown to be downregulated by ibuprofen, thereby interfering with testicular descent (Jensen et al., 2010; Kristensen et al., 2011; Maamar et al., 2017; Sharpe 2020).

Though not directly involved with the testes, it is worth briefly mentioning that neuro-morphological and neurochemical differentiation involved in characteristic male sexual behavior involve interactions between the eicosanoid pathway and steroid hormones and can be targets for NSAIDs and analgesic drugs as well. For example, induction of dendritic spines in the first few days of postnatal life in rats involves an estrogen-dependent increase in COX2 and PGE2, which maintains the density responsible for the masculinization of the male rat brain (Amateau and McCarthy 2004; Schwarz and McCarthy 2008). While the analgesic effect of acetaminophen is due to its ability to inhibit COX activity in the brain, it has been recently shown that its metabolite, *p-*aminophenol, can also cross the blood-brain-barrier and act on vanilloid and cannabinoid receptors. Therefore, it is quite possible that NSAIDs and analgesic drugs can adversely impact male reproductive development outside the testes (Ohashi et al., 2017; Ohashi and Kohno 2020).

Overall, there have been few studies on the role of COX enzymes in spermatogenesis. Nonetheless, the accumulation of evidence to support involvement of COX enzymes in the pathogenesis of testicular disease would suggest a role of the eicosanoid pathway that is significant to proper male reproductive development but the mechanism behind their involvement is relatively still unknown. Recent efforts have turned towards understanding potential effects induced by prostanoid end products due to their well-characterized roles as vital chemical messengers within the body and their involvement in inflammatory responses, the female reproduction, and embryonic development.

### 4.2 Expression of PG Synthases and Prostanoid Receptors in the Testis

Whereas studies would suggest a protective role of COX enzymes against testicular insult, reports on whether PGs have been beneficial to the testes have been controversial in the past. Some groups have determined that exogenous PGs can be harmful to the testes (Cenedella 1975; Ellis 1977), but others have found that PGs are important mediators involved in inflammatory processes behind testicular damage (Prasad et al., 1974). Since the 70s, efforts to better understand the function of the eicosanoid pathway in maintaining testicular homeostasis have been focused on understanding how different PGs signal on a cellular basis. So next, we will explore the expression of PGs in various cell types in the testes, and then dive deeper in the known roles of specific PGs in testicular development as outlined in Table 1.

**TABLE 1:** Summary of the current knowledge on the role of prostaglandins in male reproduction. Expression of prostaglandins (PGs), PG synthases and receptors in the male reproductive system in mammalian species are provided with brief summaries on their roles.

PGs	PG synthase	PG receptor	Cell type/Tissue	Species	Finding(s)	References
PGD2	L-PGDS	DP1	Testis NT2/D1 testicular cancer cell line	Human	PIG1 enhances PGD2 production, further increasing cAMP levels and SOX9 activation. RIG1-PGD2 might play a role in testicular cancer cell suppression	Wu et al. (2012)
PGD2	L-PGDS		Leydig Cells, epididymis	Bovine and human	Positive correlation between PGDS and PGD2 levels. L-PGDS found in Leydig cells; may play a role as retinoid-binding protein to maintain spermatogenesis	Tokugawa et al. (1998)
PGD2	L-PGDS		Sertoli cells and germ cells	Mouse	L-PGDS^−/−^ XY gonads showed abnormal SOX9 cellular localization pattern at GD11.5. SOX9 activates and maintains L-Pgds transcription. PGD2 involved in maintaining Sox9 expression during Sertoli cell differentiation. PGD2 is weakly expressed in germ cells	Moniot et al. (2009)
PGD2	L/H-PGDS	DP	Interstitial, Leydig cells, Mast cells	Human, Hamster	DP expression in interstitial cell clusters. L-PGDS is expressed and PDG2 is secreted by hamster Leydig cells. H-PGDS is expressed in mast cells	Schell et al. (2007)
PGD2	L/H-PGDS	DP2	Embryonic germ cells	Mouse	PGD2 acts through DP2 and is involved in germ cell differentiation. Nanos2 downregulated in L-PGDS−/− testes	Moniot et al. (2014)
PGD2	L/H-PGDS		Testes (postnatal)	Mouse	Spermatogonia apoptosis in L-PGDS−/− mice. 24% of L-PGDS−/− mice presented phenotype of unilateral cryptorchidism	Philibert et al. (2013)
PGD2	PGDS		Sertoli cell lineage	Mouse	PGDS is expressed in a similar dynamic spatio-temporal expression pattern in developing mouse gonads. Activating effect of PGD2 on Sox9 transcription	Wilhelm et al. (2007)
PGD2	PGDS		Sertoli cells and gonocytes (fetal)	Mouse	PGDS is expressed at GD11.5 and GD12.5 in male genital ridge. PGD2 produced in response to somatic masculinizing environment	Adams and McLaren (2002)
PGD2	PGES, PGIS, L-PGDS	DP1, DP2, EP2, EP4	Sertoli cells	Mouse	Expression of prostanoid receptors (DP1/2, EP2/4), and PGES, PGIS, and L-PGDS was found in GD10.5, 11.5, and 12.5 Sertoli cells and Sertoli-like NT2 cells. PGDS/PGD2 pathway induces Sox9 nuclear translocation in Sertoli cells	Malki et al. (2005)
PGD2			Juvenile Sertoli cells (SC5 cell line)	Mouse	PGD2 produced in SC5 cells. Compounds inhibiting COX activity reduced PGD2 synthesis levels measured by ELISA	Kugathas et al. (2016)
15-dPGJ2			Peritubular cells	Human	15-dPGJ2 influences expression of differentiation markers and contractability of peritubular cells, and is involved in the generation of fibrosis that occurs in tubule walls of infertile patients	Schell et al. (2010)
PGD2/15-dPGJ2			Pertibular cells	Human	15d-PGJ2 is detected in infertile patients and acts *via* reactive oxygen species to alter the phenotype of peritubular cells	Kampfer et al. (2012)
	L-PGDS		Testicular Sertoli and interstitial cells	Mouse	L-PGDS was expressed in Sertoli cells at stages VI-VIII of spermatogenic cycle (late spermatids) of the seminiferous epithelium	Gerena et al. (2000)
	L-PGDS		Seminiferous tubules (fetal, postnatal), Leydig Cells (Adult)	Mouse	In adult testis, L-PGDS is confined to Leydig cells. In neonatal testis, it is within seminiferous tubules. Expression decreases after birth and increases up to 10-fold between postnatal days 30–40	Baker and O Shaughnessy (2001)
	L-PGDS (as B-tr)		Leydig Cells	Mouse	L-PGDS (as B-tr) is expressed specifically in the interstitial space of the testis and in epithelia of the epididymis	Hoffman et al. (1996)
	L-PGDS (mRNA)		Sertoli and germ cells	Rat	PGDS is highest in the epididymis and is predominantly accumulated and expressed in the caput epididymis, likely being involved in sperm maturation	Sorrentino et al. (1998)
	L/H-PGDS	DP	Epididymis	Rat	DP is weakly expressed in the epididymis. H-PGDS and L-PGDS is expressed weakly in the epididymis	Gerashchenko et al. (1998)
	PGDS		Sertoli-Sertoli/Sertoli-germ cell junctions	Rat	PGDS increases significantly at puberty onset in relation to cell junction assembly and disassembly. It is likely acting as a carrier protein in Sertoli cells to transport molecules important for spermatogenesis (retinoic acid, retinal, T3)	Samy et al. (2000)
PGE2		EP1, EP2, EP4	Pertibular cells	Human	PGE2 is expressed in human testicular peritubular cells. EP3 was found in spermatogonia and was the only EP not expressed in peritubular cells. PGE2 elevated GDNF levels by 2-fold after 3h, and increased mRNA levels for calponin	Rey-Ares et al. (2018)
PGE2, PGF2a		EP1-4, IP, FP	Sertoli cells	Rat	Il-1B induces Sertoli cell PGE2 and PGF2a production, PGs can induce IL-1b in dose- and time-dependent but COX2 independent manner	Ishikawa and Morris (2006)
PGE2, PGI2			Sertoli cells	Rat	Sertoli cells synthesize PGE2 and PGI2 and FSH potentiates PG production and cAMP production. PGF2a may regulate germ cell development	Cooper and Carpenter (1987)
		EP and FP	Stem and Adult Leydig cells	Rat	Rat Leydig progenitors express FP, EP1, EP2, and EP4. EP1 and EP4 are expressed in adult Leydig cells	Walch et al. (2003)
PGF2a		FP	Leydig Cells	Hamster	Inhibitory effect of PGF2a on StAR and 17b-HSD expression and hCG- and LH-induced testosterone synthesis	Frungieri et al. (2006)
PGF2a			Leydig Cells	Hamster	Stimulatory effect of testosterone on PGF2a production via non-classical mechanism involving phosphorylation of ERK1/2. PGF2a may act as a brake on testicular steroidogenesis	Matzkin et al. (2009)

#### 4.2.1 Prostaglandin Synthases

Out of the several major PG synthases, PGDS has been one of the most characterized in the testis. PGDS was discovered in the mouse testis in the 1990s expressed in Leydig cells and in the epithelial cells of the epididymis (Hoffmann et al., 1996). This finding was corroborated by several studies reporting the presence of L-PGDS in the Sertoli cells during stages VI-VIII of the spermatogenic cycle, and in the interstitial cells of the mouse testes (Gerena et al., 2000), as well as measurable levels of PGD2 produced in juvenile mouse Sertoli cell line SC5 (Kugathas et al., 2016). When tracing L-PGDS expression during early murine reproductive development, Baker and O’Shaughnessy found L-PGDS localized in the tubules during fetal and postnatal testes. The synthase begins to be expressed between GD11.5–12.5 in the male genital ridge, and expression decreases after birth and upregulates approximately 10-fold between PND30-40, and in the adult, L-PGDS is found in Leydig cells (Baker and O Shaughnessy 2001; Adams and McLaren 2002). In the rat, both H- and L-PGDS were found to be expressed in the epididymis, and in Sertoli and germ cells, where they are involved in sperm maturation (Gerashchenko et al., 1998; Sorrentino et al., 1998). Like in the mouse, PGDS in the rat increases in expression from birth to adulthood, with clear increases starting in prepubertal rats when the formation of the blood-testis-barrier takes place. The role of PGDS in the assembly and disassembly of cell-cell junctions between Sertoli and Sertoli/germ cells suggests that PGDS may mediate the transport of molecules involved in spermatogenesis (Samy et al., 2000). Thus, in the mouse and rat models, PGDS may play important roles in supporting spermatogenesis.

As for the other synthases, PGES and PGIS were reported to be expressed in a mouse Sertoli-like cell line (Malki et al., 2005), and detectable levels of PGI2, PGE2, and PGF2a were found in rat Sertoli cells, which would suggest functional synthases (Cooper and Carpenter 1987; Ishikawa and Morris 2006). PGE2 is produced in human peritubular cells (Rey-Ares et al., 2018), and PGF2a is produced in hamster Leydig cells (Frungieri et al., 2006; Matzkin et al., 2009), which would indicate expression of PGES and PGF2a in non-rodent models as well.

#### 4.2.2 Prostaglandin Receptors

Expression of PG receptors have also been reported in several cell types in the male reproductive system. In the human, DP1 is expressed in a testicular cell line, NT2, suggesting that PGD2/DP1 may play a role in the suppression of carcinogenesis in the testis (Wu et al., 2012). DP2 is expressed in embryonic germ cells in mice (Moniot et al., 2014), and both DP1 and DP2 were expressed in fetal mouse Sertoli cells, as well as EP2 and EP4 (Malki et al., 2005). Rat epididymis express DP receptors (Gerashchenko et al., 1998), as well as other PG receptors: EP1-4, IP, and FP in Sertoli cells; EP, EP1-2, EP4 in Leydig cell progenitors; and EP1 and EP4 in adult Leydig cells (Walch et al., 2003; Ishikawa and Morris 2006). Hamster Leydig cells express DP and FP receptors (Frungieri et al., 2006; Schell et al., 2007). Human peritubular cells express EP1, EP2, and EP4 (Rey-Ares et al., 2018).

#### 4.2.3 Roles of PGD2 and 15d-PGJ2

PGD2 has also been studied widely to decipher its role in the testis. A series of studies have attempted to uncover its role in Sertoli cell maturation through the regulation of Sox9 expression. In the human-derived NT2 Sertoli-like cell line, PGD2 was shown to be involved in Sox9 nuclear translocation during embryonic development (Malki et al., 2005). Later findings in mice uncovered the ability of PGD2 to activate Sox9 transcription in cells fated to the Sertoli cell lineage (Wilhelm et al., 2007). In L-PGDS^−/−^ gonads of mice at GD11.5, Sox9 cellular localization was found to be altered, definitively confirming a significant role of PGD2 to regulate the differentiation of Sertoli cells during fetal development (Moniot et al., 2009).

As for mouse germ cells, PGD2 was found to be weakly expressed, but was still crucial in regulating germ cell differentiation (Moniot et al., 2009). Its production by mouse PGCs and gonocytes suggests PGD2 can be a factor released in response to a somatic masculinizing environment (Adams and McLaren 2002). In mechanistic studies utilizing mouse PGDS knockout models, Nanos2, which promotes fetal germ cell differentiation, was downregulated in a L/H-PGDS^−/−^ embryonic model (Moniot et al., 2014). Moreover, a study following the development of a postnatally derived L-PGDS^−/−^ mouse model showed that spermatogenesis was disrupted, consistent with the development of unilateral cryptorchidism in adult animals (Philibert et al., 2013).

In humans, PGDS is expressed in Leydig and peritubular cells and PGD2 levels have been detected. The PGDS/PGD2 system in human Leydig cells was hypothesized to play a role in retinoid-binding in regulation of spermatogenesis (Tokugawa et al., 1998). Expression of PGD2 in peritubular cells may be associated with greater infertility, as infertile patients were found to express changed levels of 15d-PGJ2 (metabolite of PGD2). 15d-PGJ2 is believed to be involved in altering the phenotype of human peritubular cells such that differentiation markers and contractability were affected (Schell et al., 2010; Kampfer et al., 2012). Thus, PGD2 may contribute to the generation of fibrosis that occurs in tubule walls of infertile patients.

#### 4.2.4 Roles of PGE2 and PGF2a

Though not as widely characterized as PGD2, some groups have also attempted to decipher roles of PGE2 and PGF2a in male reproductive development. In the rat, Sertoli cells were able to synthesize PGE2 and PGF2a in response to IL-1b and Follicle Stimulating Hormone (FSH), and in response PGE2 and PGF2a were able to induce IL-1b in positive feedback loop (Cooper and Carpenter 1987; Ishikawa and Morris 2006). In hamsters, PGF2a was found to stimulate testosterone production in Leydig cells when induced by human chorionic gonadotropin (HCG) and Luteinizing hormone (LH) *via* a non-classical mechanism that involves the phosphorylation of ERK 1/2. PGF2a also has a inhibitory effect on StAR and 17b-HSD, perhaps acting as a brake on testicular steroidogenesis (Frungieri et al., 2006; Matzkin et al., 2009).

PGE2 is expressed in human testicular peritubular cells, and was shown to be able to elevate GDNF levels and increase mRNA levels of calponin, supporting a role in spermatogenesis and maintenance of the testicular interstitium (Rey-Ares et al., 2018).

### 4.3 Effects of NSAIDs and Analgesic Drugs on Male Reproductive Development

The accumulation of literature suggests that members of the eicosanoid system are widely expressed in several cell types of the male reproductive system and are involved in fundamental developmental processes such as regulating germ and somatic cell differentiation, spermatogenesis, testosterone production, and more. While the specific signaling mechanisms behind these effects are still being interrogated, there is ample evidence to propose that altering normal eicosanoid synthesis with COX modulatory drugs can result in disruptions in developmental processes. This next section will discuss reported effects of NSAID and analgesic drugs when administered during the dynamic reproductive maturation that takes place during perinatal periods and review the weight of evidence in support of reported immediate and long-term reproductive effects.

#### 4.3.1 Effects of NSAIDs in Testis

Several studies have assessed the role of NSAIDs and analgesic drugs on the testis and their ability to induce characteristic reproductive birth defects. An early study conducted in mice found that aspirin (150 mg/kg) or indomethacin (1 mg/kg) inhibited the masculinization of male genitalia in GD18 embryos when exposed *in utero* from GD11-14. While administration of AA (25 or 100 mg/kg) was able to correct anti-masculinization effects such as hypospadias and shortening of the AGD, indomethacin (1 mg/kg) exposure completely blocked those protective effects when administered with AA and a positive control (Gupta and Goldman 1986). Other studies focusing on fetal exposure found similar effects. *In utero* exposure of acetaminophen (150, 250, 350 mg/kg/day) in rats from GD13 to GD21 resulted in significantly reduced AGD index (AGDi) in all dose groups, while aspirin (150, 200, 250 mg/kg/day) exposure resulted in growth retardation of the fetuses. Although there were no differences in AGD, trends towards decreases in testosterone production were observed with aspirin exposure (Kristensen et al., 2011). In a subsequent study also conducted in rats, acetaminophen at 350 mg/kg/day was dosed to dams from GD7 to GD19 and then again from PND14 to PN22. There were no differences in the AGDi in offspring, only a reduction of 1.6%, but the difference was not significant (Axelstad et al., 2014). This study contrasts with what was reported in Kristensen et al., 2011, but this difference may be attributed to the different ages of exposure, as trends towards reduction in AGDi was also observed by Alexstad et al. who reported a significant increase in the rate of retained nipples in the male offspring, suggesting an antiandrogenic activity of acetaminophen. This further highlights the importance of considering the age of exposure when assessing the risk of chemicals, due to potential differences in the reproductive susceptibility windows of the fetal versus pre-pubertal age groups.

While Gupta and Goldman saw effects of indomethacin in GD18 mice fetuses, fetal exposure to a similar dose of indomethacin (0.8 mg/kg) from GD15.5 to GD18.5 in rats resulted in very little adverse effects in the later stage fetuses or in offspring. Despite causing a significant decrease in testes weight at GD21.5, there were no alterations in testosterone levels or AGDi. The offspring did not exhibit hypospadias or cryptorchidisms and AGDi was not affected in puberty or adulthood. Interestingly, penile length was significantly decreased in the treatment group at PND25 (Dean et al., 2013). In a similar study evaluating long term effects of *in utero* exposure of ibuprofen, adult mice exposed to ibuprofen (5.6 mg/kg/day) from GD5-18 had no differences in sperm motility, viability, or response to hypoosmotic shock. There were also no differences in fertilization index or acrosomal integrity when compared to controls (Stutz and Munoz 2000).

Pre-pubertal exposure of rats (PND23 to PND43) to ibuprofen (2.4–14.3 mg/kg) compromised sperm parameters, the number of sperm, and daily sperm production. Daily sperm production was significantly decreased when rats were exposed to the highest dose of ibuprofen (14.3 mg/kg), but sperm transit time or morphology was not affected by the treatment. Furthermore, fertility was also reduced in highest dose group, including greater pre-implantation loss rates of the offspring (Barbosa et al., 2020).

Studies suggest that NSAIDs and analgesic drugs can affect testicular development in rodents. For example, several studies reported that early fetal exposure of pharmacological COX inhibitors induced an immediate reduction of AGDi in rodent models (Gupta and Goldman 1986; Kristensen et al., 2011). Other studies did not observe such an effect on the AGDi of rat offspring exposed *in utero* to these drugs, which may suggest an ability of the testes to recover by birth and subsequently, resume normal reproductive potential in puberty and adulthood (Dean et al., 2013; Axelstad et al., 2014). While fetal exposure of ibuprofen did not have long-term effects on sperm motility or viability (Stutz and Munoz 2000), pre-pubertal exposure did lead to decreased sperm production and fertility (Barbosa et al., 2020). However, no report has evaluated the immediate and long-term effects of neonatal exposure of NSAIDs, which is of interest to us due the significant plasticity of the testes during this stage of development.

#### 4.3.2 Effects of NSAIDs on Fetal and Neonatal Germ Cells

As fetal and postnatal gonocytes support lifelong spermatogenesis, studies have evaluated effects of NSAIDs and analgesic drugs directly on germ cells, particularly to determine whether they affected viability or had the ability to affect immediate and long-term spermatogenesis. Intrauterine exposure of indomethacin (0.8 mg/kg) and acetaminophen (350 mg/kg) in rats induced decreased the expression of *Oct4*, a fetal germ cell differentiation marker at ages between GD15.5 and GD17.5 (Dean et al., 2016). In GD13.5 mouse gonadal sections, *in utero* exposure of COX inhibitors (30 mg/kg/day acetaminophen, 50 mg/kg/d aspirin, 15 mg/kg/d ibuprofen) from GD10-13.5 significantly reduced the percentage of S-phase proportion of germ cells and resulted in significantly lower proliferative index. Intergenerational effects of *in utero* exposure were also observed, with the first generation exhibiting decreased sperm counts (Rossitto et al., 2019).

*In vitro* studies found similar abilities of NSAID and analgesic drugs to reduce fetal germ cell number. First and second semester human fetal testis fragments either cultured in hanging drops or used as xenografts in mice were exposed for 7 days to acetaminophen or ibuprofen, at 10 μM in organ culture, or 10 and 20 mg/kg 3 times daily respectively in mouse xenografts. In both cases, acetaminophen and ibuprofen significantly decreased fetal gonocyte numbers and proliferation compared to controls. In the same study, treatment of the human embryonal carcinoma cell line NTera2 with 10 μM acetaminophen or ibuprofen reduced the expression of pluripotency markers *Pou5f1* for both drugs, and *Tfap2c* with acetaminophen. Both drugs increased the expression of the epigenetic gene *TET1*, as well as the relative levels of epigenetic repressive regulator *H3H27me3*, while only acetaminophen significantly decreased the expression of *DNMT3b* (Hurtado-Gonzalez et al., 2018). Using a fetal testis explant gonad assay (FEGA) system, the expression of five germ cell markers were downregulated upon 48 h of ibuprofen exposure: *Pouf51, Tfap2c, Lin28a, Alpp,* and *Kit.* However, neither the morphology nor the density of germ cells were altered up to 72 h of 10^–4^ and 10^–5^ M treatment of ibuprofen (Maamar et al., 2017).

A study evaluating the *in utero* effect of the NSAID diclofenac, a derivative of phenyl acetic acid, at 0.2, 1, and 5 mg/kg/day, did not find alteration in the testes of rat offspring by histopathological analyses, nor in plasma testosterone concentrations (Ribeiro et al., 2020). However, treatment of adult rats with diclofenac sodium at 0.25, 0.50 and 1.0 mg/kg reduced total sperm count, total number of motile sperm, and sperm density in the epididymis. Upon histological testis examination, treatments with 0.50 and 1.0 mg/kg resulted in the complete arrest of spermatogenesis and shrinkage of the seminiferous tubules, whereas 0.25 mg/kg diclofenac sodium treatment primarily affected secondary spermatocytes and spermatids (Vyas et al., 2019).

In neonatal germ cells, the treatment of PND3 rat gonocytes with acetaminophen and ibuprofen at a high dose of 20 μg/ml (132 and 97 μM respectively), induced significant increases in proliferation, whereas ibuprofen at low (5 μg/ml; 24 μM) and high doses inhibited in a dose-dependent manner the RA-induced expression of *Stra8*, a gonocyte differentiation marker. Moreover, acetaminophen and ibuprofen high doses decreased *Cox2* expression, alone or combined with RA (Manku et al., 2020).

Overall, findings indicate that pharmacological COX inhibition alter differently germ cell development depending on the age at which exposure occurs. When administered during fetal development, these drugs either decrease proliferative ability (Dean et al., 2013), directly reduce germ cell number (Hurtado-Gonzalez et al., 2018), or decrease the expression of germ cell markers (Maamar et al., 2017). In contrast, neonatal exposure to these drugs increases gonocyte proliferation and decrease the ability of these cells to differentiate (Manku et al., 2020), which could lead to subsequent delays on the formation of foundational spermatogonial stem cell pool and long-term spermatogenesis.

#### 4.3.3 Effects of NSAIDs on Leydig Cells and Steroidogenesis

Most studies evaluating the effect of NSAIDs and analgesic drugs in early male reproductive development have focused on the ability of these drugs to target Leydig cells and alter androgen levels. The initial report that NSAIDs had the ability to target Leydig cells and affect hormone secretion was published 2003 when it was discovered that 1 μM indomethacin treatment significantly increased StAR protein levels in the mouse MA-10 Leydig cell line stimulated with 0.05 mM Bt2cAMP. This alteration was concomitant with an increase in progesterone production, though this was not observed with indomethacin treatment alone. Treatment with a COX2 specific inhibitor, NS398, also significantly increased StAR protein expression in addition to progesterone production in MA-10 cells stimulated with 0.05 mM Bt2cAMP, whereas no alterations were observed in StAR levels or steroid production when treated with a COX1 specific inhibitor (Wang et al., 2003).

##### 4.3.3.1 Fetal Leydig Cells

*In utero* exposure of 350 mg/kg acetaminophen in rats starting at GD13.5 resulted in reduced AGDi in GD21.5 fetuses, and mRNA expression of key enzymes of the steroidogenic pathway (*Cyp11a1, Cyp17a1*) were significantly decreased, though there was no change in Leydig cell number or size (van Den Driesche et al., 2015). In a separate study, *in utero* exposure of rats to 10–60 mg/kg of ibuprofen from GD15 to PND21 was found to lower volumes of Leydig cell nuclei by PND90, with a concomitant decrease in testosterone levels in the highest dose group. LH and FSH levels were similar between groups, and no alterations in spermatogenesis or Sertoli cell dynamics were observed (da Silva Balin et al., 2020). When fetal rat testes at GD14.5 were exposed to acetaminophen (1 μM) or aspirin (1 and 10 μM) for 24–72 h *ex vivo*, 1 uM acetaminophen reduced testosterone production and dose dependent reductions were observed with aspirin as well, with a significant reduction at the 10 μM dose (Kristensen et al., 2011). This study was corroborated with doses up to 100 μM, showing similar effects. Indomethacin at 10 μM decreased fetal rat testosterone production, but no alterations in Insulin-like hormone 3 (INSL3) were observed (Kristensen et al., 2012). However, when pregnant mouse dams were gavaged with 50 or 150 mg/kg/day of acetaminophen, there were no abnormalities in the gonads of the male offspring when they examined germ cell markers, Leydig cell morphology, or spermatogenesis, despite finding a reduction in AGDi at 10 weeks old after *in utero* exposure of acetaminophen (Holm et al., 2015).

##### 4.3.3.2 Juvenile and Peripubertal Leydig Cells

In rats exposed to ibuprofen during juvenile and peripubertal stages of development, 2.4 and 7.2 mg/kg/day doses resulted in reduced testosterone levels, while the 7.2 mg/kg/day dose presented increased FSH and altered LH levels. Furthermore, all treatment groups exhibited decreased Leydig cell number and significantly reduced Leydig cell volume in the 2.4 mg/kg/day treatment group (Barbosa et al., 2020). In human adult testis explants exposed to 10^–4^ M aspirin, 10^–5^ M indomethacin, and 10^–5^ M and 10^–4^ M acetaminophen, testosterone secretion was decreased, while INSL3 expression was reduced by both aspirin concentrations and 10^–4^ M indomethacin after 24 h, but the number of Leydig cells was increased by 10^–4^ M aspirin and 10^–5^ M indomethacin. In addition, no effects on gross morphology of the testes were observed (Albert et al., 2013). In a study of testes from young men xenografted in mice hosts, testosterone was inhibited by treating the host mice with ibuprofen at 10^–4^ M at 24 h, and 10^–5^ M at 48 h. Furthermore, ibuprofen inhibited all steroids from pregnenolone down to testosterone, which corresponded with decreases in gene expression involved in testicular steroidogenesis, except *Cyp19a1* (Kristensen et al., 2018)*.*

##### 4.3.3.3 Studies From Human-Derived Models

Using the FEGA system, dose-dependent decreases in testosterone were observed in fetal human testis cultures after 24 h of exposure to 10^–4^ and 10^–5^ M ibuprofen, at GW 8 to 9, but not in younger or older fetal testes. Human fetal testis (GW 10-12) xenografted mice exposed for 48 h to ibuprofen presented decreased expression of steroidogenic genes *Cyp11a1, Cyp17a1,* and *Hsd17b3*, and decreased INSL3 levels after 72 h of exposure. However the treatments had no effect on testosterone production in these conditions (Maamar et al., 2017). These findings are in contrast to that of another study, in which three times daily of 20 mg/kg acetaminophen exposure in human fetal testes (GW 14-20) xenograft mice resulted in significantly reduced testosterone levels compared to vehicle-exposed mice (van Den Driesche et al., 2015). This was confirmed in a subsequent experiment in which 7-days *ex vivo* exposure of 350 mg/kg acetaminophen on human fetal explants reduced overall testosterone levels.

A study evaluating mixtures of ketoconazole, BPA, valproic acid, and theophylline found a dose-dependent pattern of declining testosterone levels in human fetal testes explants (GW10-12). However, despite alterations in testosterone levels there were no morphological signs of impairments or significant changes in cleaved caspase 3 expression (Gaudriault et al., 2017). The effects of several analgesic drugs (acetaminophen, aspirin, indomethacin, and ketoconazole at doses 10^–4^ to 10^–7^ M) on *ex vivo* human fetal testes (GW 7-12) organ cultures resulted in unique alterations. Ketoconazole treatment significantly decreased testosterone and INSL3 levels were significantly decreased, whereas indomethacin and aspirin stimulated testosterone levels. While no alteration in testosterone level was found with acetaminophen or its metabolite, decreases in INSL3 levels were observed (Mazaud-Guittot et al., 2013).

Human fetus explant studies suggest decreases in testosterone levels with exposure to NSAIDs and analgesic drugs, particularly acetaminophen, when dosed between GW10-20, seemingly when targeting the latter duration of masculinizing programming window in humans (GW8-14). However, the complexity lies when considering the impact of ages of fetuses, doses, exposure levels, and the type of NSAID or analgesic used in the study on steroidogenic effects. Thus, future studies aimed at evaluating a wide range of compounds at several ages groups are important in gaining an overall better picture of the effects of these drugs on steroidogenesis.

In an *in vitro* model of steroidogenic cells, the NCI-H295R human adrenocortical cell line, INSL3 levels were decreased with exposure to both doses of aspirin and 10^–4^ M indomethacin, but no effects were observed with ketoconazole or acetaminophen treatment on INSL3 production (Albert et al., 2013). Interestingly, in another study, exposure of acetaminophen to up 1,000 μM increased pregnenolone and decreased hormone levels downstream from progesterone. Treatment of dipyrone (100, 314, and 1,000 μM) on human derived H295R cells reduced concentrations of testosterone and was concomitant with an increase in progesterone and induction of CYP21 activity. However, intrauterine dipyrone (50, 100, or 200 mg/kg/day) did not result in testosterone reduction in contrast to DEHP at 750 mg/kg/day, which was used as a positive control (Passoni et al., 2018).

Overall, these studies strongly suggest that NSAIDs and analgesic drugs can target Leydig cells and alter their ability to regulate hormone levels, particularly testosterone, as reported in *ex vivo* (Kristensen et al., 2012; Albert et al., 2013; van Den Driesche et al., 2015; Gaudriault et al., 2017; Maamar et al., 2017), *in utero* (da Silva Balin et al., 2020), and *in vitro* (Passoni et al., 2018) studies. In support of these effects, several studies reported alterations of steroidogenic enzymes (Wang et al., 2003; van Den Driesche et al., 2015; Maamar et al., 2017; Kristensen et al., 2018; Passoni et al., 2018). There is ample evidence to support the conclusion that COX inhibitors can affect normal Leydig cell function and impact steroidogenesis. Despite the findings presented here, there is a gap in the understanding of the link between prostaglandins and steroidogenesis. Use of NSAIDs and analgesic drugs may only partially involve prostaglandin inhibition in the Leydig cell and thereby may be disrupting steroidogenesis through additional mechanisms such as by inhibiting steroidogenic enzymes or inducing oxidative stress (Kristensen et al., 2011; Kristensen et al., 2012; Dean et al., 2013; Kristensen et al., 2016; Bauer et al., 2021). Cell-type specific effects of these drugs on other testicular cell types must be considered as well, as the normal function of the testes relies on the coordinated efforts of all the cell types involved.

#### 4.3.4 Effects of NSAIDs on Sertoli Cells

While there have been many reports on detrimental the effects of NSAID and analgesic drugs on the development of germ and Leydig cells, significantly less findings were reported on Sertoli cells. *In utero* exposure of either low (3.6 mg/kg/day), medium (9 mg/kg/day), or high (18 mg/kg/day) diclofenac sodium from GD15-21, rat pups at PND7 showed significant decreases in Sertoli cell at the medium and high drug doses. Interestingly, seminiferous tubules did not exhibit lumens, and the tubules consisted only of spermatogonia and Sertoli cells. Spermatogonia were also significantly reduced with the medium and high doses, with many cells exhibiting pycnotic nuclei and remnants of dead cells. However, no significant differences were observed in AGD or nipple retention (Arslan et al., 2016).

*Ex vivo* ibuprofen treatment of 10^–5^ and 10^–4^ M in human fetal testes (GW 8–12) explants dependently inhibited Anti-Müllerian hormone (AMH) levels. This is supported by decreases in gene expression of *Amh* and *Sox9* with the 10^–5^ M ibuprofen dose at 24 and 48 h (Maamar et al., 2017). Aspirin treatment of human fetal testes (GW 7–12) cultures induced AMH production in Sertoli cells, whereas trends in increases were observed with acetaminophen and ibuprofen treatment, and ketoconazole inhibited AMH production. However, none of the treatments altered Sertoli or germ cell ratio (Mazaud-Guittot et al., 2013).

Despite some effects observed in rats with *in-utero* exposure of NSAID diclofenac sodium on Sertoli cell number and the morphology of the tubules, there were no alterations in Sertoli cell ratio observed in human testes explants. Other studies that evaluated effect of NSAIDs on the testes found no changes in Sertoli cell number (van Den Driesche et al., 2015; Maamar et al., 2017; Rossitto et al., 2019; da Silva Balin et al., 2020), despite some decreases in AMH protein and mRNA levels and *Sox9* levels (Maamar et al., 2017; Kristensen et al., 2018). Therefore, further studies will need to be conducted on Sertoli cell models to gain a more complete understanding of the effect of NSAIDs and analgesic drugs on early male reproductive development.

#### 4.3.5 Effects of NSAIDs on Testicular Macrophages

No studies on the effects of NSAIDs and analgesics drugs on testicular macrophages have been reported in the literature. However, it is well established that macrophages are targets of pharmaceutical COX inhibitors and these drugs have been found to affect TNFα release, nitric oxide production, and proliferation (Plant and Zeleznik 2014). In the testis, macrophages are intimately involved in cord formation, germ cell development, Leydig cell function, and the maintenance of testicular privilege (DeFalco et al., 2014; Plant and Zeleznik 2014; Meinhardt et al., 2018). Studies have reported that prostaglandins, in particular PGE2 and PGI2, can affect the polarization status of testicular macrophages, from M1 to M2 phenotypes. Their presence in testis, together with PGF2a and PGD2, and changes in testicular Cox2 levels in testis during inflammation suggest that exposure to acetaminophen or NSAIDs could alter testicular macrophages, suggesting a potential effect of these drugs on testicular macrophages functions (Meinhardt et al., 2018). Therefore, it is likely the immunomodulatory effects of NSAID and analgesic drugs can impact the abilities of testicular macrophages to carry out such functions related to maintaining testicular homeostasis, highlighting the need for more studies in human.

## 5 Epidemiological Evidence

In support of experimental findings, epidemiological studies in humans have also reported adverse effects in early male reproductive development with exposure to NSAID and analgesic drugs. TDS conditions such as cryptorchidism, hypospadias, and reduced AGD were positively associated with maternal use of COX inhibitors at varying stages of pregnancy.

### 5.1 Cryptorchidism

A 2010 study evaluating infant sons of mothers that were using acetaminophen, ibuprofen, or acetylsalicylic acid during pregnancy found that drug use during the first and second trimesters were associated with increased cryptorchidism and orchiopexy, and exposure throughout all 3 trimesters were associated with increased risk as well. Use during a single trimester or during the male programming window at gestational week (GW)8–14 was only weakly associated with cryptorchidism in infants. Exposure to ibuprofen or acetylsalicylic acid alone was not associated with cryptorchidism, neither was exposure of to a combination of drugs. Interestingly, when drugs were used consistently for longer durations (5–8 weeks) or when they were used during and beyond the 4-weeks male programming window, this resulted in cryptorchidism in offspring (Jensen et al., 2010). This study is supported by a 2012 prospective cohort study which followed the growth of infants to early adulthood in the Netherlands. Mothers were asked to report their use of mild analgesics (NSAIDs, acetaminophen, painkillers like aspirin) during pregnancy at 12 gestational weeks, 20 gestational weeks, and 30 gestational weeks. Amongst the mothers with cryptorchid sons, 33.3% reported use of mild analgesics during pregnancy, and amongst those with sons with hypospadias, 31.8% reported use of mild analgesics. However, there were still 29.9% of women who report use of mild analgesics with healthy sons. The study did find that the use of mild analgesics during periconception was not associated with cryptorchidism or hypospadias, but that use between GW14-22 was associated with increased risk of cryptorchidism, even after adjusting for individual compound use. Use during GW20-32 was not associated with cryptorchism or hypospadias (Snijder et al., 2012). Lastly, a prospective birth cohort study conducted in Denmark found that mothers who use mild analgesics during pregnancy had a significant association with giving birth to a cryptorchid baby boy. This was significant for ibuprofen and acetylsalicylic acid and trended in the same direction but was not significant for acetaminophen. For mothers using multiple analgesics or using them for more than 2 weeks during pregnancy, the risk was even higher. The highest risk for cryptorchid babies was found in mothers who were using multiple compounds for more than 2 weeks (Kristensen et al., 2011). A study conducted between 2003 and 2006 assessed women who were exposed to analgesics (aspirin, acetaminophen, ibuprofen) within the first 2 trimesters of pregnancy or throughout the entire pregnancy. They reported a 4.6% frequency of undescended testes in offspring compared with 2.9% of unexposed offspring, as well as an odds ratio of 1.2 for women who used analgesics during the first two trimesters, which was similar to those who were exposed throughout the pregnancy (odds ratio = 1.5) (Philippat et al., 2011).

### 5.2 Hypospadias

Using data from the National Birth Defects Prevention Study (2007–2011), Interrante et al. evaluated associations between NSAID use and birth defects including hypospadias. They found that approximately 80% of women reported using analgesics during pregnancy, and among the COX inhibitor user group, acetaminophen and ibuprofen use during pregnancy was significantly associated with hypospadias, in addition to a variety of other birth defects including gastroschisis and spina bifida (Interrante et al., 2017). However, the ability of acetaminophen to cause hypospadias is somewhat controversial as another assessment of the Danish National Birth Cohort of mothers who had been exposed to acetaminophen use during the first trimester of pregnancy did not find any association between acetaminophen use and higher prevalence of congenital abnormalities (Rebordosa et al., 2008). However, a positive correlation between ibuprofen use and the development of hypospadias is supported by another study that evaluated the associations between maternal use of common medications and herbal remedies during periconceptual period and early pregnancy. The authors reported an association with analgesic use (aspirin, meperidine HCl, and ibuprofen), but only ibuprofen use was significantly associated with hypospadias, after adjusting for confounding factors in the population, such as maternal age, ethnicity, education, BMI, etc. (Lind et al., 2013).

### 5.3 Reduced AGD

In 2017, a prospective birth study was conducted in which pregnant women were asked about their medication use of acetaminophen and NSAIDs during GW10-27 and at GW28. Lind et al. found no association between acetaminophen and reduced AGD in boys 3 months after birth, but exposure to both acetaminophen and other NSAIDs was associated with a shorter AGD. There was no statistically significant association between acetaminophen use and reduced AGD in boys, but a strong tendency was observed when acetaminophen was used during the second trimester of pregnancy. Simultaneous exposure to multiple pain killers did result in a significant association. Interestingly, in mothers who reported analgesic use but did not specify when during pregnancy, analgesic use was positively associated with boys presenting a smaller penile widths compared to non-exposed boys (Lind et al., 2017). In a prospective cohort study conducted in the United Kingdom in 2016, mothers self-reported medication uses during pregnancy, and infants’ AGD, penile length, and testicular descent were assessed at 0, 3, 12, 18, and 24 months of age. Acetaminophen exposure during GW8-14 was associated with a reduced AGD, and this reduction is consistent with those measured at birth through 24 months of age. Penile length or testicular descent were not significantly associated with acetaminophen exposure during pregnancy, nor was there an association between maternal exposure and cryptorchidism in this study (Fisher et al., 2016).

### 5.4 Long-Term Male Reproductive Effects

While NSAID and analgesic exposure have been found to cause adverse effects in infants, studies are starting to evaluate long-term effects in pubertal youth and in adults. A study using data derived from the Danish National Birth Cohort evaluated the association between pre- and perinatal exposure of maternal use of acetaminophen and pubertal effects. While some associations between intrauterine exposure of greater than 12 weeks and a shift towards later Tanner pubic hair stages were observed, there were no strong associations with male pubertal development (Ernst et al., 2019). In adults, a prospective study of couple’s fecundity found that men with high urinary acetaminophen had an increased time to pregnancy (Smarr et al., 2016). Regular NSAID users also had lower serum testosterone (17% compared to non-users) and albumin levels compared to non-users, but had no difference in serum AMH, sex-hormone binding globulin, or inhibin B (Halpern et al., 2020). Another study correlating regular NSAID use of adult men throughout life and male reproductive parameters found no associations between drug use and semen quality or male reproductive hormones. Interestingly, in this study, use of NSAIDs was correlated with significantly higher levels of testosterone as well as use of combination drugs (acetaminophen, NSAIDs, and antihistamines) (Høyer et al., 2017).

## 6 Non-Rodent Studies

Non-rodent models have been studied to assess whether eicosanoid pathway genes are expressed and whether their responses to COX inhibitors are similar to that of humans. In the bull, PGDS activity was detected in bull seminal plasma, as well as the presence of PGD2. PGDS was also present in the luminal fluids collected from the testis and the epididymis. L-PGDS is believed to be involved in the rapid conversion of PGD2 to 15-d-PGJ2 in the bull, and likely functions as a carrier protein for transport across the blood-testis-barrier into the seminal plasma (Gerena et al., 1998).

Zebrafish models have many advantages for the study of developmental processes due to their low cost, small size, and similarity of developmental processes to mammalian models. In a study evaluating the characterization of the zebrafish and chicken homologues of mammalian L-PGDS, Grozzer et al. found that exon/intron junctions were conserved and that the enzyme bound lipophilic molecules like lipocalin gene family proteins (Fujimori et al., 2006). Zebrafish gonads at 42- and 90- days post fertilization (dpf) were found to express *ptgs1* and two *ptgs2* genes, *ptgs2a* and *ptgs2b*, and their expressions were higher in testes than ovaries. *Pgds* expression was higher in 90-dpf testes and *pges* expression was high in both 42- and 90-dpf ovaries. Testes produced higher PGE2 and PGD2 levels, and higher expression of PGE2 and PGD2 receptors as well. 20-dpf zebrafish exposed to 30 uM meloxicam for 6 days had increased expression of male-specific genes *Sox9a* and *dmrt1a,* while female specific genes were downregulated. Exposure of a PGD2 analog, BW-245C, induced expression of *Sox9a* after 24 and 48 h in testes explants and in juvenile zebrafish (Pradhan and Olsson 2014). Exposure of BW-245C at 70 dpf resulted in male-biased sex ratios, suggesting that PGD2 influences male sexual development, similar to what was reported in the mammalian studies (Malki et al., 2005; Wilhelm et al., 2005). Furthermore, it was discovered that expression of *Pgds* in the gonads is localized to Sertoli cells. Initiation of *Pgds* expression seems to be initiated between 1 and 2 dpf. *Pges*, on the other hand, was expressed both in male and female gonads (Jørgensen et al., 2010). When zebrafish embryos were exposed to 0.1, 1, 10 and 50 mg/L NSAIDs (acetylsalicylic acid, ketoprofen, indomethacin, naproxen, ibuprofen, nimesulide, celecoxib) from 0 to 6 dpf, genes from the PG pathway were downregulated following to exposure from all NSAIDs (*ptgs1*, *ptgs2a*, and *ptgs2b*), despite being Cox1 or Cox2 selective. *Pges* was not altered, but *Pgds* was upregulated for only the Cox-2 selective inhibitors (nimesulide and celecoxib). Ibuprofen and naproxen resulted in 22 and 18% increase in the male population, respectively, while Cox-2 selective inhibitors resulted in 31.6% and 26.6% increase in the male population (Bereketoglu et al., 2020). This is consistent with the 2014 study which found that meloxicam, another Cox2 selective inhibitor, was able to induce male-biased populations in zebrafish (Pradhan and Olsson 2014).

In a study conducted on another fish species, the juvenile rainbow trout, who were fed once a day with salicylate (100 mg/kg) incorporated into the feed, acute ACTH-mediated cortisol production was significantly inhibited, reflecting alteration of adrenal steroidogenesis. Gene expression of *StAR* and *PBR*, which are involved in cholesterol transport, were also downregulated in salicylate exposed fish. No significant difference was observed in 11b-hydroxylase and P450scc expression. Similar results were obtained from *in vitro* studies in cells exposed to ibuprofen and acetaminophen in addition to salicylate (Gravel and Vijayan 2006). Effect of AA on goldfish testes was evaluated in the presence and absence of hormonal stimulation, which caused a stimulation of testosterone production in both conditions of hormonal stimulation over the course of 20-h incubation. Incubation of indomethacin and ibuprofen resulted in reductions of AA-induced testosterone production, while PGE2, PGI2, and PGF2a caused an increase in testosterone production, whereas PGD2 had no effect (Wade et al., 1994). This work was corroborated with a later study that continued to investigate whether the steroidogenic activity of fatty acids was mediated through conversion to cyclooxygenase products. AA at 400 uM stimulated testosterone production, and this effect was blocked by indomethacin. Testis pieces were incubated with PGE1, PGE2, and PGE3, which all induced testosterone production. Studies in both the trout and goldfish suggest that the eicosanoid pathway can modulate steroidogenesis in fish testes (Wade et al., 1994).

In the chicken, *cPGDS* was found to be expressed in embryos starting on day 6.5 (stage 30) in the male genital ridge only. By day 8.5, both *cPGDS* and *cSOX9* are co-expressed by Sertoli cells within the testicular cords. Some germ cells (*cSOX9-*) were found to also express *cPGDS*, similarly to what has been shown in mammalian studies. Expression of *cPGDS* increases in a linear fashion from day 6.0 to day 8.0 (Moniot et al., 2008). In male chicken gonadal explants, PGD2 was found to activate *cSOX9* expression when visualized with *in situ* hybridization, similar to what was shown in the mouse and zebrafish (Malki et al., 2005; Wilhelm et al., 2005; Pradhan and Olsson 2014).

In insects, the *Chironomus ripaius* (harlequin fly) were treated with 0, 0.1, 1, 10 and 100 μg/L of ibuprofen for 24, 48, 72, and 96 h. Met, a receptor for Juvenile hormone (JH) which regulates of metamorphosis and reproductive processes, was downregulated by ibuprofen exposure, despite JH expression not being altered by the drug (Muñiz-González 2021).

Studies in non-rodent models have shown presence of eicosanoid pathway activity in species such as the bull, fish, chicken, and insects. In fish, the PGDS and PGD2 axis is important for sexual differentiation, even though PGE2 is the major prostanoid found in both the zebrafish and the rainbow trout. COX inhibitors are able to target the eicosanoid pathway in these models, and alter sex ratios (Pradhan and Olsson 2014; Bereketoglu et al., 2020) or hormone synthesis (Wade et al., 1994; Gravel and Vijayan 2006). The ability of NSAIDs to affect development processes have been observed with the chicken (Moniot et al., 2008) and the fly as well (Muñiz-González 2021). Overall, studies in non-rodent species have found similar results as what has been reported in rodent models, with the eicosanoid pathway having wide-ranging roles in reproductive development, steroidogenesis, and maturation of male gonads.

## 7 Conclusion

**FIGURE 2:**
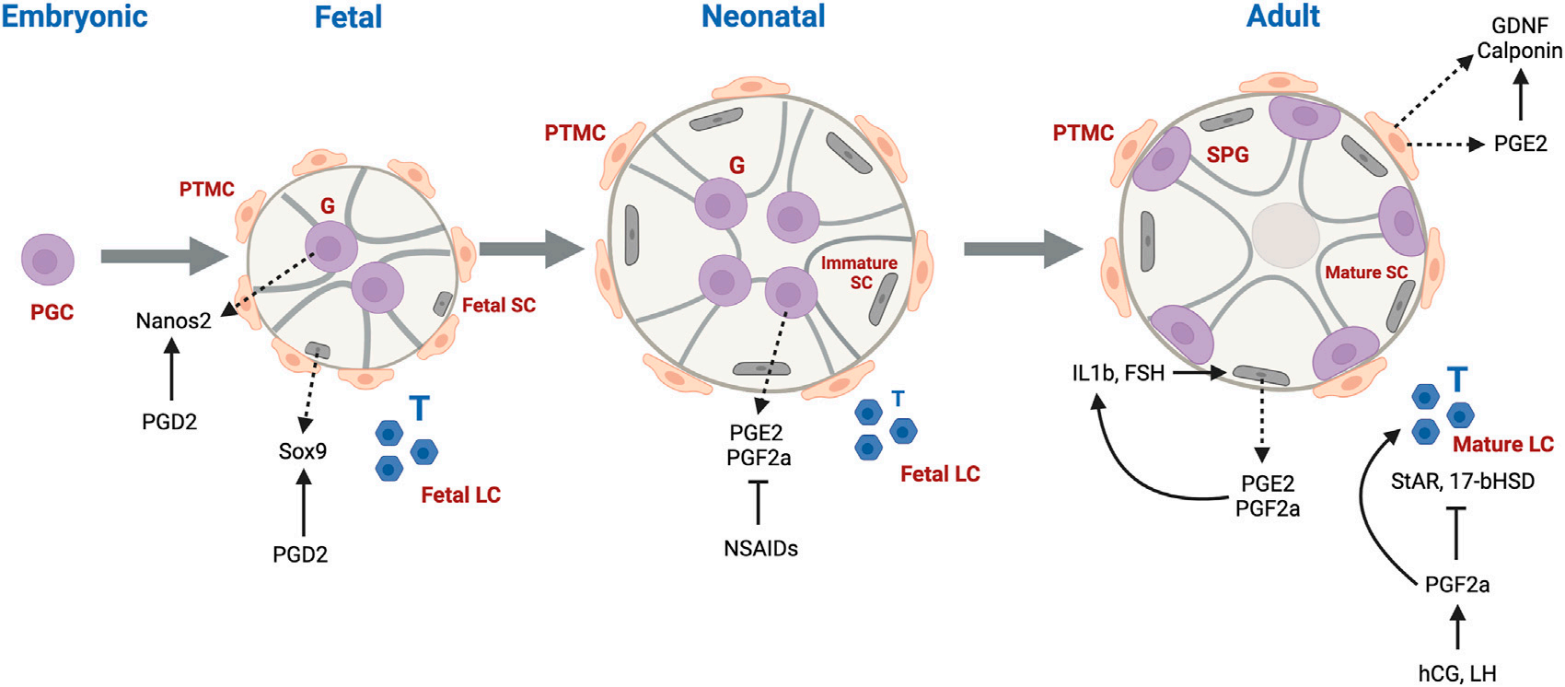
Schematic illustration of the production and roles of prostaglandins (PGs) in mammalian testis from development to adulthood. PGD2 induces mouse germ cell differentiation *via* Nanos2 and activates Sox9 produced by immature mouse Sertoli cells. PGE2 and PGF2a are produced by neonatal rat gonocytes and levels are inhibited by NSAIDs. PGE2 and PGI2 synthesized by mature Sertoli cells can activate FSH and Il1b in rats. In turn, Il1b can stimulate Sertoli cells to release more PGs. In hamsters, hCG- and LH-stimulated PGF2a has an inhibitory effect on StaR and 17b-HSD synthesis. In human PTMCs, PGE2 can elevate GDNF and Calponin levels. Abbreviations: PGC: Primordial Germ Cell, G: Gonocyte, LC: Leydig Cell, Fetal SC: Fetal Sertoli Cell, Immature SC: Immature Sertoli Cell, Mature SC: Mature Sertoli Cell, SPG: Spermatogonia, PTMC: Peritubular Myoid Cell. T: Testosterone. Dotted arrow: factors produced by specific cell types; (↑): positive effects; (T): negative effect.

Overall, multiple studies suggest that the eicosanoid pathway is expressed throughout the testes and mechanistic studies have shown substantial PG involvement in processes of germ cell development and steroidogenesis, such that when PG synthesis is disrupted with pharmacological COX inhibitors, these processes are the ones primarily affected. What is currently known about the involvement of PGs in the regulation of testicular cell types from the embryonic stage of development to adulthood in various species is summarized in Figure 2. Laboratory-based studies reporting adverse effects such as cryptorchidism, hypospadias, and reduced AGD were corroborated by epidemiological studies of infants exposed to NSAIDs and analgesic drugs *in utero*. Similar findings were observed in non-rodent studies as well, which further supports a critical role of the eicosanoid pathway in early male reproductive development. However, more research is necessary to correlate these mechanistic findings to adverse reproductive disorders observed in epidemiological studies. These limitations may be due to a lack of innovative tools to measure direct effects of exposures in offspring (Liew and Ernst 2021) and the lack of studies conducted to evaluate exposures beyond the pregnancy and throughout development. Furthermore, these effects are particularly important to explore in premature babies and neonates, who are often long-term consumers of NSAIDs and analgesic drugs (Kristensen et al., 2016). Despite these limitations, sufficient evidence supports that the eicosanoid pathway is an important regulatory pathway involved in male reproductive development, and scientists are just beginning to unveil the immediate and long-term adverse effects that exposure to pharmacological COX inhibitors can have on the developing male reproductive system.

## Data Availability

The original contributions presented in the study are included in the article/Supplementary Materials, further inquiries can be directed to the corresponding author.

## Glossary

TDS: testicular dysgenesis syndrome
17β-HSD: 17β-hydroxysteroid dehydrogenase
AA: arachidonic acid
ACTH: adrenocorticotropic hormone
AGD: anogenital distance
AGDi: anogenital distance index
AMH: anti-müllerian hormone
BPA: bisphenol A
COX: cyclooxygenase
DPF: days post fertilization
EDCs: endocrine disrupting compound
FEGA: fetal gonad assay
FSH: follicle stimulating hormone
GD: gestational day
GPCR: G-coupled protein receptor
GW: gestational week
HCG: human chorionic gonadotropin
HTPCs: human testicular peritubular cells
IL: interleukin
INSL3: insulin-like hormone 3
JH: juvenile hormone
LH: luteinizing hormone
LPS: lipopolysaccharide
NSAID: non-steroidal anti-inflammatory drug
PBR: peripheral-type benzodiazepine receptor
PGCs: primordial germ cells
PGDS/PTGDS: prostaglandin D synthase
PGES: prostaglandin E synthase
PGIS: prostaglandin I synthase
PGs: prostaglandins
PLA2: phospholipase A2
PND: postnatal day
RA: retinoic acid
SCOS: sertoli-cell-only syndrome
SSC: spermatogonial stem cell
StAR: steroidogenic acute regulatory protein
TGCTs: testicular germ cell tumors
TXA2: thromboxane
